# Supplementary material for: Pyruvate Dehydrogenase Kinase 1 inhibition mediated oxidative phosphorylation enhancement in cartilage promotes osteoarthritis progression
Source: BMC Musculoskelet Disord. 2023 Jul 20;24:597. doi: 10.1186/s12891-023-06585-6 (PMC10357736; doi:10.1186/s12891-023-06585-6)
Supplement: Supplementary file 1 — Additional file 1. [file 12891_2023_6585_MOESM1_ESM.zip › MF.Gsea.1653627316242/GOMF_HSP90_PROTEIN_BINDING.html]

Details for gene set GOMF\_HSP90\_PROTEIN\_BINDING[GSEA]

|  || Dataset | 55457\_collapsed\_to\_symbols.cls.cls #OA\_versus\_normal.cls.cls #OA\_versus\_normal\_repos |
| Phenotype | cls.cls#OA\_versus\_normal\_repos |
| Upregulated in class | normal |
| GeneSet | GOMF\_HSP90\_PROTEIN\_BINDING |
| Enrichment Score (ES) | -0.42119303 |
| Normalized Enrichment Score (NES) | -1.5694767 |
| Nominal p-value | 0.011475409 |
| FDR q-value | 0.15830614 |
| FWER p-Value | 0.999 |
Table: GSEA Results Summary

  

Fig 1: Enrichment plot: GOMF\_HSP90\_PROTEIN\_BINDING      
 Profile of the Running ES Score & Positions of GeneSet Members on the Rank Ordered List

  

| SYMBOL | TITLE | RANK IN GENE LIST | RANK METRIC SCORE | RUNNING ES | CORE ENRICHMENT || 1 | STUB1 | STIP1 homology and U-box containing protein 1 [Source:HGNC Symbol;Acc:HGNC:11427] | 345 | 0.726 | 0.0278 | No |
| 2 | NR3C1 | nuclear receptor subfamily 3 group C member 1 [Source:HGNC Symbol;Acc:HGNC:7978] | 674 | 0.599 | 0.0474 | No |
| 3 | PPEF2 | protein phosphatase with EF-hand domain 2 [Source:HGNC Symbol;Acc:HGNC:9244] | 2249 | 0.333 | -0.0500 | No |
| 4 | NOD2 | nucleotide binding oligomerization domain containing 2 [Source:HGNC Symbol;Acc:HGNC:5331] | 3154 | 0.235 | -0.1026 | No |
| 5 | CDK5 | cyclin dependent kinase 5 [Source:HGNC Symbol;Acc:HGNC:1774] | 3400 | 0.211 | -0.1057 | No |
| 6 | TELO2 | telomere maintenance 2 [Source:HGNC Symbol;Acc:HGNC:29099] | 3650 | 0.190 | -0.1108 | No |
| 7 | CDC37 | "cell division cycle 37, HSP90 cochaperone [Source:HGNC Symbol;Acc:HGNC:1735]" | 3810 | 0.175 | -0.1100 | No |
| 8 | GUCY1B1 | guanylate cyclase 1 soluble subunit beta 1 [Source:HGNC Symbol;Acc:HGNC:4687] | 3962 | 0.159 | -0.1097 | No |
| 9 | MAPT | microtubule associated protein tau [Source:HGNC Symbol;Acc:HGNC:6893] | 3976 | 0.158 | -0.0989 | No |
| 10 | PPID | peptidylprolyl isomerase D [Source:HGNC Symbol;Acc:HGNC:9257] | 5029 | 0.071 | -0.1754 | No |
| 11 | ERN1 | endoplasmic reticulum to nucleus signaling 1 [Source:HGNC Symbol;Acc:HGNC:3449] | 5658 | 0.019 | -0.2228 | No |
| 12 | GBP1 | guanylate binding protein 1 [Source:HGNC Symbol;Acc:HGNC:4182] | 6490 | -0.042 | -0.2843 | No |
| 13 | NUP62 | nucleoporin 62 [Source:HGNC Symbol;Acc:HGNC:8066] | 6800 | -0.063 | -0.3035 | No |
| 14 | TSC2 | TSC complex subunit 2 [Source:HGNC Symbol;Acc:HGNC:12363] | 6946 | -0.075 | -0.3091 | No |
| 15 | HDAC6 | histone deacetylase 6 [Source:HGNC Symbol;Acc:HGNC:14064] | 7833 | -0.143 | -0.3673 | No |
| 16 | PACRG | parkin coregulated [Source:HGNC Symbol;Acc:HGNC:19152] | 8128 | -0.165 | -0.3777 | No |
| 17 | CYP1A1 | cytochrome P450 family 1 subfamily A member 1 [Source:HGNC Symbol;Acc:HGNC:2595] | 8195 | -0.171 | -0.3700 | No |
| 18 | KPNB1 | karyopherin subunit beta 1 [Source:HGNC Symbol;Acc:HGNC:6400] | 8285 | -0.177 | -0.3636 | No |
| 19 | STIP1 | stress induced phosphoprotein 1 [Source:HGNC Symbol;Acc:HGNC:11387] | 8559 | -0.198 | -0.3699 | No |
| 20 | AHSA1 | activator of HSP90 ATPase activity 1 [Source:HGNC Symbol;Acc:HGNC:1189] | 8706 | -0.210 | -0.3655 | No |
| 21 | PTGES3 | prostaglandin E synthase 3 [Source:HGNC Symbol;Acc:HGNC:16049] | 8802 | -0.217 | -0.3565 | No |
| 22 | TTC4 | tetratricopeptide repeat domain 4 [Source:HGNC Symbol;Acc:HGNC:12394] | 9116 | -0.241 | -0.3627 | No |
| 23 | AHR | aryl hydrocarbon receptor [Source:HGNC Symbol;Acc:HGNC:348] | 9263 | -0.253 | -0.3551 | No |
| 24 | UNC45A | unc-45 myosin chaperone A [Source:HGNC Symbol;Acc:HGNC:30594] | 9267 | -0.253 | -0.3363 | No |
| 25 | USP19 | ubiquitin specific peptidase 19 [Source:HGNC Symbol;Acc:HGNC:12617] | 10017 | -0.323 | -0.3702 | No |
| 26 | EIF2AK3 | eukaryotic translation initiation factor 2 alpha kinase 3 [Source:HGNC Symbol;Acc:HGNC:3255] | 10025 | -0.324 | -0.3464 | No |
| 27 | CYP2E1 | cytochrome P450 family 2 subfamily E member 1 [Source:HGNC Symbol;Acc:HGNC:2631] | 10988 | -0.428 | -0.3890 | Yes |
| 28 | FKBP6 | FKBP prolyl isomerase family member 6 (inactive) [Source:HGNC Symbol;Acc:HGNC:3722] | 11173 | -0.454 | -0.3691 | Yes |
| 29 | HSF1 | heat shock transcription factor 1 [Source:HGNC Symbol;Acc:HGNC:5224] | 11189 | -0.456 | -0.3360 | Yes |
| 30 | RPS3 | ribosomal protein S3 [Source:HGNC Symbol;Acc:HGNC:10420] | 11245 | -0.463 | -0.3054 | Yes |
| 31 | AHSA2P | "activator of HSP90 ATPase homolog 2, pseudogene [Source:HGNC Symbol;Acc:HGNC:20437]" | 11278 | -0.468 | -0.2727 | Yes |
| 32 | KDR | kinase insert domain receptor [Source:HGNC Symbol;Acc:HGNC:6307] | 11454 | -0.496 | -0.2489 | Yes |
| 33 | TSC1 | TSC complex subunit 1 [Source:HGNC Symbol;Acc:HGNC:12362] | 11575 | -0.511 | -0.2198 | Yes |
| 34 | CHORDC1 | cysteine and histidine rich domain containing 1 [Source:HGNC Symbol;Acc:HGNC:14525] | 11689 | -0.531 | -0.1886 | Yes |
| 35 | SLC12A2 | solute carrier family 12 member 2 [Source:HGNC Symbol;Acc:HGNC:10911] | 11744 | -0.540 | -0.1522 | Yes |
| 36 | HIF1A | hypoxia inducible factor 1 subunit alpha [Source:HGNC Symbol;Acc:HGNC:4910] | 11833 | -0.556 | -0.1172 | Yes |
| 37 | CSNK2A1 | casein kinase 2 alpha 1 [Source:HGNC Symbol;Acc:HGNC:2457] | 11895 | -0.570 | -0.0791 | Yes |
| 38 | ARNTL | aryl hydrocarbon receptor nuclear translocator like [Source:HGNC Symbol;Acc:HGNC:701] | 12120 | -0.625 | -0.0495 | Yes |
| 39 | NPAS2 | neuronal PAS domain protein 2 [Source:HGNC Symbol;Acc:HGNC:7895] | 12345 | -0.689 | -0.0151 | Yes |
| 40 | PPP5C | protein phosphatase 5 catalytic subunit [Source:HGNC Symbol;Acc:HGNC:9322] | 12548 | -0.773 | 0.0274 | Yes |
Table: GSEA details [plain text format]

  

Fig 2: GOMF\_HSP90\_PROTEIN\_BINDING      
 Blue-Pink O' Gram in the Space of the Analyzed GeneSet

  

Fig 3: GOMF\_HSP90\_PROTEIN\_BINDING: Random ES distribution      
 Gene set null distribution of ES for **GOMF\_HSP90\_PROTEIN\_BINDING**
